# A randomized clinical trial evaluating choroidal blood flow and morphology after conventional and pattern scan laser panretinal photocoagulation

**DOI:** 10.1038/s41598-018-32487-y

**Published:** 2018-09-20

**Authors:** Yuji Mikoshiba, Takeshi Iwase, Yoshitaka Ueno, Kentaro Yamamoto, Eimei Ra, Hiroko Terasaki

**Affiliations:** Department of Ophthalmology, Nagoya University Graduate School of Medicine, Nagoya, Japan

## Abstract

We prospectively investigated the changes in choroidal blood flow and morphology after panretinal photocoagulation (PRP) in 39 eyes with severe nonproliferative diabetic retinopathy (S-NPDR). Seventeen eyes underwent PRP by conventional laser and 22 eyes underwent pattern scan laser (PASCAL). The choroidal blood flow was assessed by laser speckle flowgraphy, and the subfoveal choroidal thickness (SFCT) was measured with optical coherence tomography before and 1, 4, 8, and 12 weeks after the two types of PRP treatments. The choroidal mean blur rate (MBR) at the macular region was significantly reduced to 86.4% of the baseline level in the conventional laser group and 85.7% in the PASCAL group at Week 12 (*P* = 0.001, *P* < 0.001, respectively). The SFCT was significantly increased at 1 week following PRP but it was significantly reduced at Week 8 *(P* = 0.001, *P* < 0.001, respectively) in both groups. The differences in the ratio of the MBR and the SFCT was not significant between the conventional laser and PASCAL groups at any time after PRP. The results suggest that appropriate PRP treatments even by the PASCAL method will reduce the choroidal blood flow and the choroidal morphological components.

## Introduction

Diabetic retinopathy is one of the main causes of severe vision loss in the industrialized world. Studies have demonstrated that panretinal photocoagulation (PRP) treatments were associated with good long-term visual acuity in most patients with proliferative diabetic retinopathy (PDR)^1–3^ and severe non-PDR (S-NPDR)^4^.

It has also been reported that there is a significant decrease in the retinal blood flow following PRP in eyes affected by S-NPDR or PDR^5–8^. In addition, studies have shown that there is a significant reduction in choroidal blood flow after PRP^8–10^. Savage *et al*. reported that the choroidal blood flow determined by pulsatile ocular blood flow is decreased in eyes with laser-treated PDR^9^. It has also been reported that there is a significantly greater reduction in the choroidal blood flow in eyes with laser-treated S-NPDR than in non- treated S-NPDR eyes and normal eyes^8^. On the other hand, Takahashi *et al*. reported that PRP might increase the choroidal blood flow in eyes with S-NPDR^11^. It has still not been definitively determined whether PRP will alter the choroidal blood flow probably because of the differences in the measurement methods, instruments, measured regions, and stage of the disease process.

Laser speckle flowgraphy (LSFG; Softcare Co., Ltd., Fukutsu, Japan) is a noninvasive quantitative method for determining ocular blood flow without the use of contrast agents^12,13^, based on the changes in the speckle pattern of laser light reflected from the fundus of the eye. The blood flow rate can be determined by the changes in the pattern of scatter in the vessels, and the rate is expressed by the mean blur rate (MBR)^12^. LSFG uses a laser illuminating light with a relatively long wavelength of 830 nm which has greater tissue penetrance thus enabling blood flow recordings from the choroid.

There have been morphological studies that demonstrated that the choroidal thickness is decreased for a long time after PRP^8,14,15^, although some studies showed a transient increase in the very early post-PRP treatment period, e.g., 1 week after the PRP^15,16^. However, it has not been conclusively determined whether the choroidal thickness is altered after PRP. Furthermore, Okamoto *et al*. reported that there was a significant positive correlation between the percentage reduction of the SFCT and choroidal MBR at 12 weeks after PRP^17^. However, the effect of laser treatment on the choroidal blood flow and its relationship to the choroidal thickness is still controversial.

Blumenkranz *et al*. described a semi-automated patterned scanning laser (PASCAL) treatment technique^18^ that allowed a rapid application of multiple laser spots in an array with short pulse durations which decreased the width and the axial extent of the retinal burns of the RPE and outer retinal layer^19^. Therefore, the PASCAL treatment protocol would have an advantage over conventional laser therapy on the choroidal blood flow and the thickness. However, a search of Pubmed did not extract any publications on the changes in the choroidal blood flow and the thickness after PRP by the PASCAL method. Thus, a comparison of the benefits of PASCAL and conventional laser therapy on the choroidal blood flow in addition to its relationship of the changes of the choroidal blood flow and the choroidal thickness needs to be determined for eyes with DR.

Thus, the purpose of this study was to determine the changes in the choroidal blood flow and morphology after conventional laser and PASCAL treatment in patients with S-NPDR, and to compare the changes between the conventional laser and PASCAL treatment. To accomplish this, the choroidal blood flow was determined by LSFG and the subfoveal choroidal thickness (SFCT) and the choroidal area was measured by analysing the SD-OCT images to evaluate the morphological changes following PRP.

## Results

### Patient demographics

A total of 46 patients with type II diabetes and S-NPDR with no macula edema were screened and 43 were enrolled. Three patients did not meet inclusion criteria. Among the 43 patients, 21 were assigned to the conventional laser group and 22 to the PASCAL group. Of the 21 patients in the conventional group, 4 patients dropped out during the PRP treatment. In the end, 17 eyes of 17 patients in the conventional laser group and 22 eyes of 22 patients in the PASCAL group were studied. In addition, 15 another patients who had S-NPDR and did not receive laser treatment were compared with patients who received laser treatments as a control group. In the conventional laser group, there were 12 men and 5 women whose mean age was 55.5 ± 11.5 years, the mean best-corrected visual acuity (BCVA) was 0.08 ± 0.10 logMAR units, and the mean duration of the diabetes was 12.6 ± 7.6 years. In the PASCAL group, there were 12 men and 10 women whose mean age was 55.6 ± 11.8 years, the mean BCVA was 0.09 ± 0.12 logMAR units, and the mean duration of the diabetes was 11.9 ± 8.1 years. In the control group, there were 12 men and 3 women whose mean age was 58.8 ± 8.1 years, the mean BCVA was 0.08 ± 0.11 logMAR units, and the mean duration of the diabetes was 12.7 ± 8.0 years. There were no significant differences in the systemic and ocular variables among the three groups (Table 1).

**Table 1:** Clinical characteristics of subjects.

Characteristics	Conventional laser	PASCAL laser	Control	*p* - value
n	17	22	15	—
Age (years)	55.5 ± 11.5	55.6 ± 11.8	58.8 ± 8.1	0.651
Males/Females	12/5	12/10	12/3	0.417
BCVA (Log MAR)	0.08 ± 0.10	0.09 ± 0.12	0.08 ± 0.11	0.963
Intraocular pressure (mmHg)	15.2 ± 2.7	15.0 ± 2.8	14.3 ± 1.9	0.621
Axial length (mm)	23.87 ± 0.73	23.91 ± 1.23	24.02 ± 1.25	0.928
Foveal thickness (μm)	227.4 ± 19.7	230.1 ± 28.4	236.7 ± 13.6	0.509
Subfoveal choroidal thickness (μm)	293.2 ± 55.0	291.6 ± 41.1	272.4 ± 47.1	0.414
Systolic blood pressure (mmHg)	128.8 ± 32.1	131.6 ± 28.0	129.5 ± 16.8	0.925
Diastolic blood pressure (mmHg)	75.8 ± 18.7	78.3 ± 14.1	75.5 ± 10.5	0.773
Ocular perfusion pressure (mmHg)	47.1 ± 14.5	49.0 ± 12.8	47.9 ± 6.6	0.880
Heart rate (bpm)	81.8 ± 8.0	79.8 ± 14.3	82.0 ± 12.6	0.621
Duration of diabetes (year)	12.6 ± 7.6	11.9 ± 8.1	12.7 ± 8.0	0.951
Insulin/oral antidiabetic drugs	10/7	12/10	9/6	0.984
HbA1c (%)	8.4 ± 1.9	9.3 ± 2.5	8.0 ± 1.7	0.215
Body mass index (kg/m2)	24.5 ± 5.0	24.2 ± 6.5	24.1 ± 3.1	0.986
Total cholesterol (mg/dL)	166.1 ± 43.1	175.5 ± 60.5	141.6 ± 33.7	0.280
Triglycerides (mg/dL)	130.3 ± 48.1	134.1 ± 72.8	125.0 ± 56.9	0.850
Serum creatinin (mg/dL)	0.89 ± 0.29	0.75 ± 0.21	0.86 ± 0.18	0.327
eGFR (mL/min/1.73 m2)	69.3 ± 24.6	78.9 ± 27.3	64.1 ± 22.2	0.154

BCVA: best corrected visual acuity, LogMAR: logarithm of the minimum angle of resolution.

The mean number of the photocoagulation burns was 1524 ± 157 in the conventional laser group and 4959 ± 582 in the PASCAL group. None of the patients developed any adverse effects related to the PRP. One eye had a temporal macular edema greater than 300 µm and less than 400 µm after the PRP treatment in each of the conventional laser group and the PASCAL group. Both eyes did not undergo any treatment for the macular edema, e.g. anti-VEGF treatment or steroid treatment. The stage of the DR had not progressed at 12 weeks after the PRP as determined by the FA findings. The difference in the BCVA before the PRP and that throughout the post-PRP period was not significant (Table 2).

**Table 2:** Change in variable parameters with time.

	Parameter	Before PRP	Week 1	Week 4	Week 8	Week 12	*p*-value
Conventional laser	BCVA (Log MAR)	0.08 ± 0.10	0.12 ± 0.10	0.11 ± 0.11	0.09 ± 0.0.11	0.09 ± 0.10	0.185
	Choroidal MBR (AU)	7.08 ± 2.02	6.52 ± 2.08	6.16 ± 1.51	6.18 ± 1.23	6.08 ± 1.44	0.001
	Foveal thickness (μm)	227.4 ± 19.7	240.4 ± 30.0	250.6 ± 44.7	252.7 ± 38.3	259.7 ± 51.2	<0.001
	SFCT (μm)	293.2 ± 54.9	304.6 ± 55.9	291.2 ± 55.6	286.3 ± 56.7	275.7 ± 57.1	<0.001
	Choriocapillaris layer thickness (μm)	59.2 ± 11.0	61.9 ± 12.1	58.5 ± 12.4	57.6 ± 11.1	53.9 ± 11.2	<0.001
	Choroidal area (mm^2^)	0.435 ± 0.096	0.459 ± 0.101	0.436 ± 0.098	0.426 ± 0.097	0.409 ± 0.092	<0.001
	Luminal area (mm^2^)	0.284 ± 0.067	0.296 ± 0.066	0.284 ± 0.066	0.277 ± 0.065	0.270 ± 0.065	<0.001
	Stromal area (mm^2^)	0.151 ± 0.034	0.163 ± 0.040	0.152 ± 0.039	0.149 ± 0.041	0.138 ± 0.038	<0.001
	IOP (mmHg)	15.1 ± 2.7	15.1 ± 2.6	14.1 ± 2.8	13.4 ± 1.9	13.7 ± 1.9	0.020
	MOPP (mmHg)	47.1 ± 14.5	48.4 ± 13.5	45.1 ± 11.1	48.3 ± 7.7	48.3 ± 8.5	0.834
PASCAL	BCVA (Log MAR)	0.09 ± 0.12	0.11 ± 0.12	0.11 ± 0.12	0.10 ± 0.11	0.09 ± 0.13	0.230
	Choroidal MBR (AU)	7.21 ± 1.94	6.72 ± 1.70	6.46 ± 1.96	6.66 ± 1.77	6.18 ± 1.45	<0.001
	Foveal thickness (μm)	230.5 ± 28.3	239.6 ± 39.1	244.7 ± 40.2	246.4 ± 31.7	245.0 ± 37.9	0.001
	Choriocapillaris layer thickness (μm)	57.7 ± 7.8	61.0 ± 7.9	57.1 ± 6.7	56.1 ± 7.5	54.8 ± 8.2	<0.001
	SFCT (μm)	291.6 ± 41.0	305.2 ± 44.6	290.7 ± 42.2	282.1 ± 39.8	277.0 ± 44.1	<0.001
	Choroidal area (mm^2^)	0.421 ± 0.076	0.448 ± 0.081	0.410 ± 0.065	0.408 ± 0.080	0.399 ± 0.084	<0.001
	Luminal area (mm^2^)	0.282 ± 0.054	0.294 ± 0.048	0.272 ± 0.048	0.271 ± 0.053	0.267 ± 0.058	<0.001
	Stromal area (mm^2^)	0.139 ± 0.032	0.153 ± 0.038	0.137 ± 0.029	0.136 ± 0.035	0.131 ± 0.034	<0.001
	IOP (mmHg)	15.0 ± 2.8	13.6 ± 2.8	13.7 ± 3.0	14.3 ± 2.5	14.5 ± 2.8	0.010
	MOPP (mmHg)	48.9 ± 12.7	47.2 ± 8.9	47.0 ± 9.0	46.2 ± 8.9	46.2 ± 10.5	0.557
Control	BCVA (Log MAR)	0.08 ± 0.11	0.06 ± 0.08	0.07 ± 0.11	0.07 ± 0.12	0.06 ± 0.08	0.542
	Choroidal MBR (AU)	6.97 ± 1.82	7.12 ± 2.17	7.01 ± 1.71	6.99 ± 1.75	7.11 ± 1.96	0.854
	Foveal thickness (μm)	236.7 ± 13.6	243.3 ± 19.3	237.3 ± 19.5	242.7 ± 19.8	239.6 ± 18.1	0.270
	SFCT (μm)	272.4 ± 47.1	269.6 ± 52.7	273.1 ± 41.3	272.0 ± 40.8	272.7 ± 49.6	0.670
	Choriocapillaris layer thickness (μm)	57.8 ± 11.5	58.6 ± 14.3	58.5 ± 11.5	58.1 ± 13.1	58.3 ± 12.3	0.313
	Choroidal area (mm^2^)	0.379 ± 0.061	0.379 ± 0.068	0.384 ± 0.062	0.372 ± 0.054	0.382 ± 0.065	0.765
	Luminal area (mm^2^)	0.254 ± 0.036	0.259 ± 0.035	0.264 ± 0.035	0.259 ± 0.029	0.257 ± 0.039	0.792
	Stromal area (mm^2^)	0.125 ± 0.029	0.120 ± 0.026	0.128 ± 0.030	0.121 ± 0.023	0.125 ± 0.031	0.556
	IOP (mmHg)	14.3 ± 1.9	14.6 ± 2.6	13.8 ± 2.4	13.7 ± 2.0	14.3 ± 1.9	0.479
	MOPP (mmHg)	47.9 ± 6.6	47.5 ± 9.2	47.1 ± 9.9	47.7 ± 7.3	47.6 ± 7.9	0.997

AU: arbitrary unit, BCVA: best corrected visual acuity, LogMAR: logarithm of the minimum angle of resolution.

SFCT: subfoveal choroidal thickness, IOP intraocular pressure, MOPP: mean ocular perfusion pressure.

### Changes in choroidal MBR

**Figure 1:**
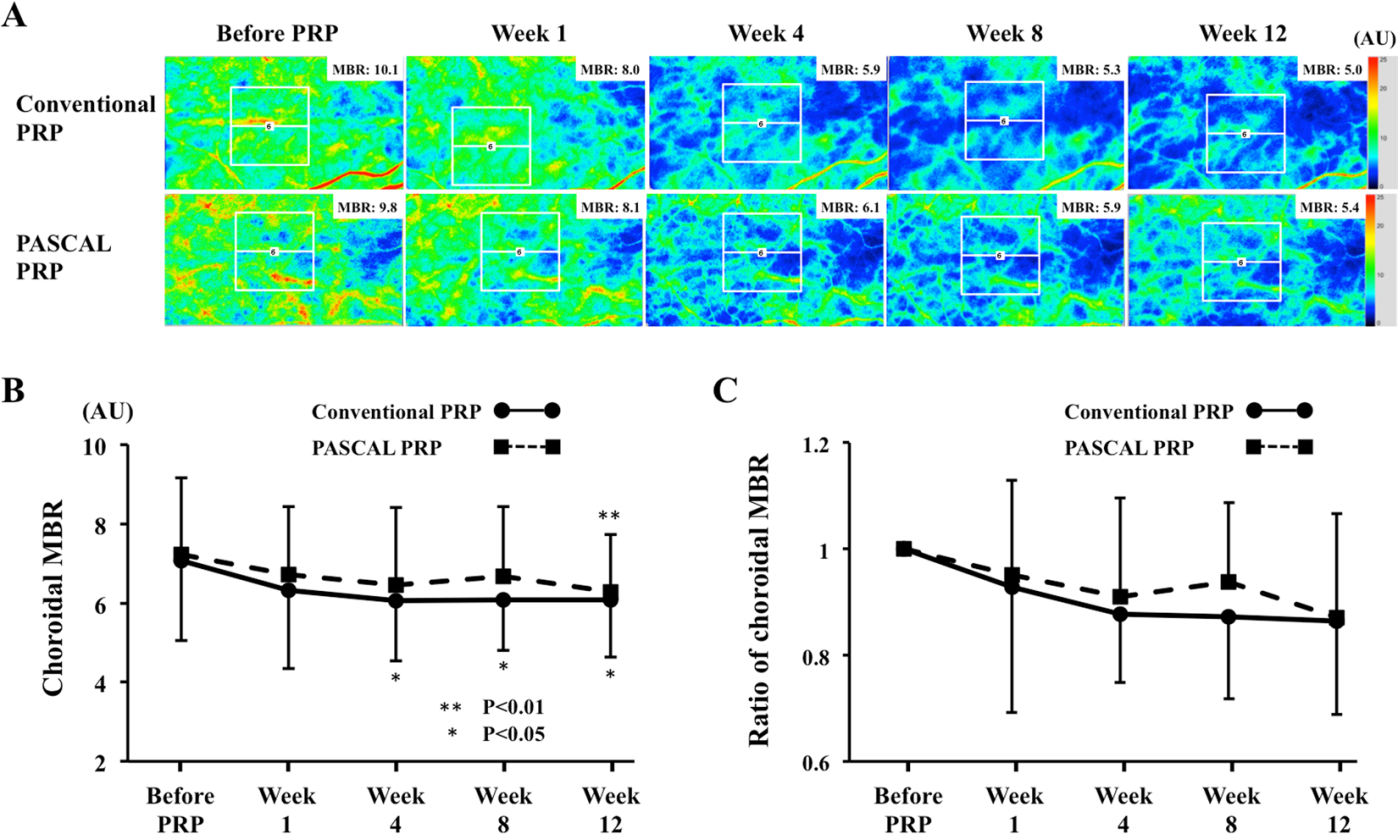
Representative composite colour maps of the mean blur rate (MBR) as determined by laser speckle flowgraphy (LSFG) in eyes with S-NPDR before, and 1, 4, 8, and 12 weeks after panretinal photocoagulation (PRP) performed by conventional laser and pattern scan laser (PASCAL) treatment at the macula (**A**). The red colour indicates a high MBR, and the blue colour indicates a low MBR. To measure the choroidal MBR, the center of a square (250 × 250 pixels; 6.31° × 6.31°) was set at the fovea. There was a significant reduction in the choroidal MBR in both the conventional laser and the PASCAL PRP treatment groups (**B**) after the PRP. The ratio of the choroidal MBR to the baseline was not significantly different between the conventional laser and the PASCAL treatment groups throughout the post-PRP period (**C**).

In the conventional laser group, the mean macular choroidal MBR was 7.1 ± 2.0 arbitrary units (AU) before PRP, 6.5 ± 2.1 (91.5%) AU at 1 week, 6.2 ± 1.5 AU (87.8%) at 4 weeks, 6.2 ± 1.2 AU (87.2%) at 8 weeks, and 6.1 ± 1.4 AU (86.4%) at 12 weeks following PRP (Table 2). The macular choroidal MBR were significantly reduced at 4, 8, and 12 weeks after the PRP (Week 4, *P* = 0.028; Week 8, *P* = 0.026; Week 12, *P* = 0.021; Fig. 1B).

In the PASCAL group, the mean choroidal MBR was 7.2 ± 1.9 AU before the PRP, 6.7 ± 1.7 AU (93.1%) at 1 week, 6.5 ± 1.9 AU (90.9%) at 4 weeks, 6.7 ± 1.7 AU (93.8%) at 8 weeks, and 6.2 ± 1.4 AU (86.9%) at 12 weeks following the PRP. The macular choroidal MBR was significantly reduced only at 12 weeks after PRP (*P* = 0.001).

In the control group, the macular choroidal MBR did not significantly change during follow-up period.

The ratio of MBR to the baseline was not significantly different between the conventional laser group and the PASCAL group at any time (Fig. 1C).

### Morphological changes in choroid

**Figure 2:**
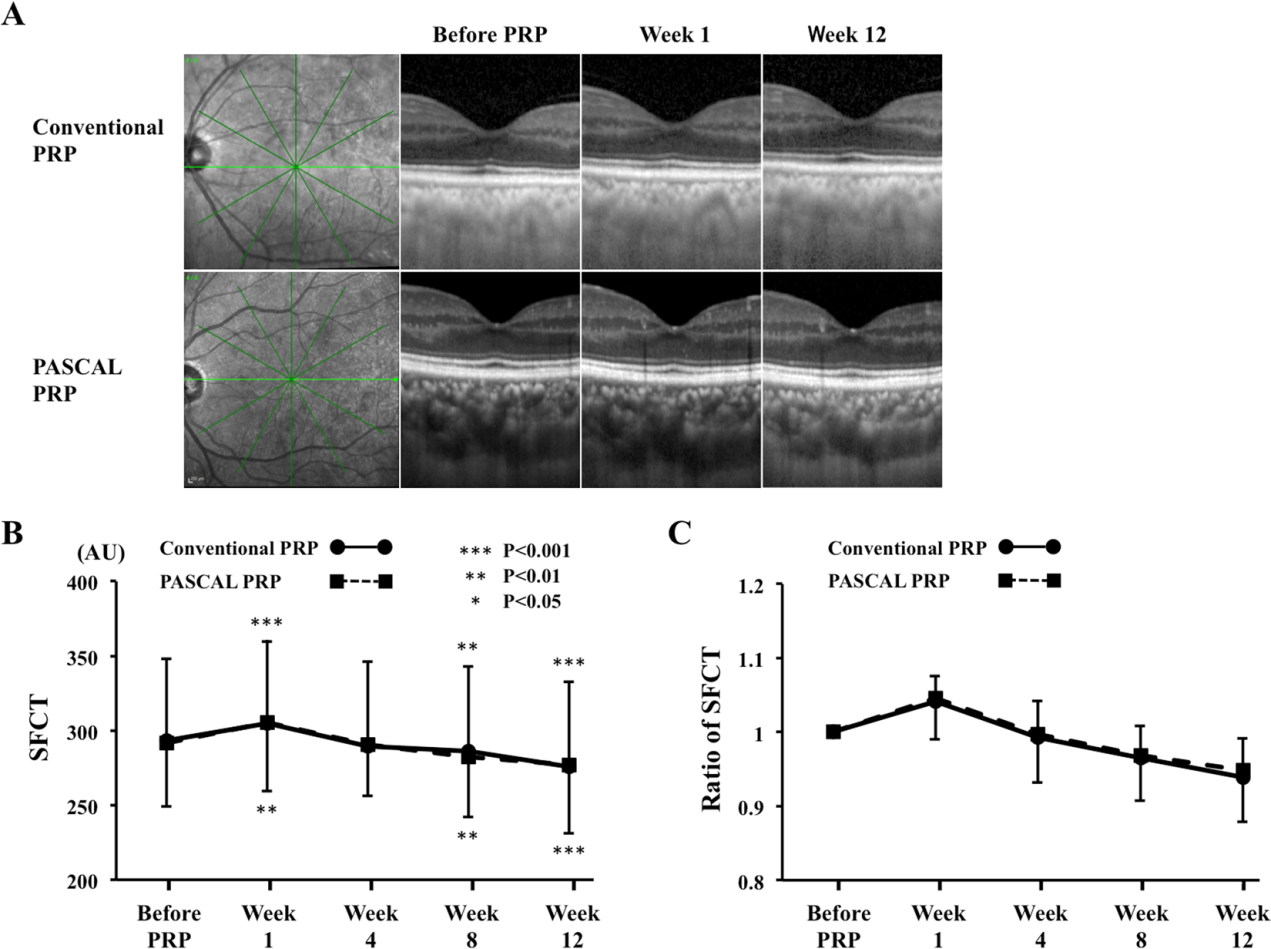
Representative horizontal SD-OCT images of an eye with S-NPDR before and 1, 4, 8, and 12 weeks after PRP with the conventional laser and the PASCAL at the macula (**A**). The SFCT at Week 1 was significantly thicker than that before PRP in the conventional laser and the PASCAL group, and the SFCT was significantly decreased in the both groups at 8 and 12 weeks after PRP (**B**). The trend of the changes in the SFCT was similar in both groups, and there was no significant difference between the groups (**C**).

**Figure 3:**
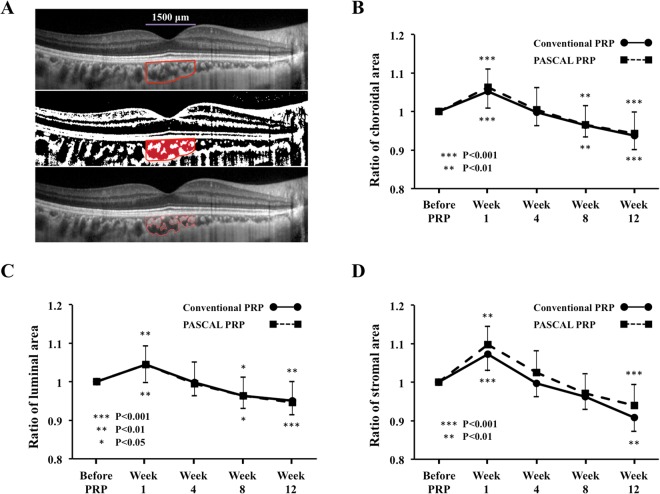
Representative binarized image of the choroidal area in an enhanced depth imaging (EDI) optical coherence tomographic (OCT) image (**A**). The area of interest of the choroid is outlined (top). The EDI-OCT image is converted to a binary image using the ImageJ software. The rectangle surrounded by the red line was excised, and the dark areas were traced by the modified Niblack method (middle). The binarized image and the margin of the traced area were merged which demonstrates that the traced area is consistent with the dark areas of the choroidal areas of the OCT image (bottom). The ratio of the choroidal area (**B**) the luminal area (**C**) and the stromal area (**D**) to the baseline was not significantly different between the conventional laser and the PASCAL groups. ***P < 0.001, **P < 0.01, *P < 0.05.

In the conventional laser group, the mean SFCT was significantly thicker at 1 week following PRP (*P* < 0.001) but was significantly thinner at Week 8 (*P* = 0.006) and Week 12 (*P* < 0.001; Table 2, Fig. 2). The SFCT at Week 12 was 93.9% of the baseline thickness. The choriocapillary layer thickness was significantly thicker at 1 week following PRP (*P* = 0.003) but was significantly thinner at Week 8 (*P* = 0.044) and Week 12 (*P* < 0.001) (Table 2). The mean choroidal, luminal, and stromal areas were significantly increased from that at the baseline at 1 week following PRP (*P* < 0.001, *P* = 0.008, *P* = 0.001, respectively), but the mean choroidal and luminal areas were significantly decreased at Week 8 *(P* = 0.017, *P* = 0.001) and Week 12 *(P* = 0.001, *P* < 0.001), and the stromal area was significantly decreased at Week 12 (*P* < 0.001; Fig. 3).

In the PASCAL group, the morphological changes of the choroid beneath the macula were similar to that in the conventional laser group. The mean SFCT was significantly thicker than the baseline at 1 week following PRP (*P* < 0.001), but it was significantly thinner than that at the baseline at Week 8 (*P* = 0.001) and Week 12 (*P* < 0.001). The SFCT at Week 12 was 94.8% of the baseline thickness. The choriocapillary layer thickness was significantly thicker at 1 week following PRP (*P* < 0.001) but was significantly thinner at Week 8 (*P* < 0.001) and Week 12 (*P* < 0.001). The mean choroidal, luminal, and stromal areas were significantly increased at 1 week following PRP (*P* < 0.001, *P* = 0.001, *P* < 0.001, respectively), while the mean choroidal and luminal areas were significantly decreased at Week 8 (*P* = 0.001, *P* < 0.001) and Week 12 *(P* < 0.001, *P* < 0.001), and the stromal area was significantly decreased at Week 12 (*P* = 0.007).

In the control group, there were no significant changes in the SFCT, the choriocapillary layer thickness, the choroidal, luminal, and stromal areas during the follow-up period.

The ratio of the SFCT, choroidal, luminal, and stromal areas to the baseline was not significant different between the conventional laser group and the PASCAL group at any time.

The repeatability of the measurements between the observers was excellent with an ICC of 0.990 (confidence interval [CI] 0.984–0.993) for the SFCT, 0.995 (0.993–0.996) for the choroidal area, 0.995 (0.993–0.996) for the luminal area, and 0.997 (0.995–9.997) for the stromal area. These findings indicate that the measurement of the SFCT and the choroidal area determined by binalization method had good repeatability.

### Correlations between choroidal MBR and other parameters

Pearson correlation coefficient analyses showed that the correlation between the ratio of choroidal MBR at Week 12 to the baseline to other parameters, e.g., the ratio of SFCT at Week 12 to the baseline and the number of laser burns were not significant (Table 3). The trend of the change in choroidal MBR was not significantly correlated with that in the SFCT, the choriocapillary layer thickness, BCVA, IOP, and MOPP (Table 4).

**Table 3:** Results of Pearson’s correlation coefficient between the reduction ratio of choroidal MBR at Week 12 to the baseline and other variables.

Variables	Conventional laser		PASCAL	
	*r*	*p*-value	*r*	*p*-value
Ratio of SFCT at Week 12 to the baseline	0.011	0.967	0.058	0.808
Axial length	−0.289	0.260	0.07	0.769
Laser burns	−0.050	0.848	−0.221	0.350
Duration of diabetes	0.256	0.376	0.078	0.801
HbA1c	−0.446	0.11	−0.229	0.452
Heart rate	−0.023	0.931	−0.403	0.078
Age	0.405	0.151	0.03	0.923

SFCT: subfoveal choroidal thickness.

**Table 4:** Correlation between choroidal MBR and other factors with time.

Factor	Conventional laser				PASCAL	
	Coefficient	95% Confidence interval	*p*-value	Coefficient	95% Confidence interval	*p-*value
SFCT	−0.002	−0.011 to 0.006	0.566	−0.002	−0.013 to 0.008	0.625
Foveal thickness	−0.004	−0.015 to 0.007	0.439	−0.002	−0.014 to 0.009	0.699
Choriocapillaris layer thickness	−0.001	−0.016 to 0.013	0.865	−0.01	−0.015 to 0.013	0.89
BCVA	−1.730	−5.539 to 2.079	0.368	−1.157	−4.373 to 2.059	0.477
IOP	−0.120	−0.293 to 0.052	0.167	−0.092	−0.221 to 0.037	0.158
MOPP	−0.003	−0.083 to 0.032	0.864	0.01	−0.027 to 0.047	0.609

SFCT: subfoveal choroidal thickness, BCVA, best corrected visual acuity, IOP: intraocular pressure.

MOPP: mean ocular perfusion pressure.

## Discussion

Our results showed that there was a gradual decrease of the choroidal MBR for at least 12 weeks following the PRP by the conventional laser or the PASCAL protocols in eyes with S-NPDR. In addition, PRP caused a significant increase of the SFCT at 1 week following the completion of the PRP, but the SFCT was significantly reduced after Week 8 in the both groups. There were no significant differences in the ratio of the choroidal MBR and the SFCT to the baseline values between the conventional laser and the PASCAL groups throughout the post-PRP period. In addition, there were no significant correlations in the trend between the choroidal MBR and the other variables.

The results of earlier studies showed that the changes in the choroidal blood flow in the macula area were inconsistent after conventional PRP^8–11,17,20–23^. Takahashi *et al*. reported that the subfoveal choroidal blood flow measured by laser Doppler flowmetry was increased at one month after PRP^11^. On the other hand, two studies reported that the choroidal blood flow was significantly reduced in PRP-treated eyes compared with that of untreated eyes as measured by pulsatile ocular blood flow and color Doppler imaging 6 months to 2 years after PRP^9,10^. It has been reported that the choroid blood flow determined by LSFG at the macula was significantly slower by 70% of that of normal subjects^8^. In addition, Okamoto *et al*. reported that the choroidal MBR determined by LSFG decreased significantly to 87.5% of the baseline values at 4 weeks and 86.0% at 12 weeks^17^. Our results indicated that the choroidal MBR was significantly decreased to 87.8% of the baseline at 4 weeks and 86.4% at 12 weeks of the baseline value in the conventional laser group. These findings are in good agreement with those of Okamoto *et al*.

The findings on the SFCT in the early post-PRP period varied in previous studies. We found that the SFCT was increased in both groups at 1 week after PRP which corroborates previous studies that PRP is associated with increases in the SFCT at 1 week after completion of the PRP^15,16^. However, the SFCT was not significantly different from the baseline thickness at 4 weeks after the PRP. These results indicate that the SFCT is increased at the very early period after PRP but the thickening of the SFCT is resolved by 4 weeks after the PRP.

The exact mechanisms causing the early increase in SFCT after PRP has not been determined but some evidence can be obtained from our results and the results of previous studies. Our results showed that the luminal area determined by the binarization method and the SFCT were increased at 1 week after PRP. This may be due to the vasodilation of the choroidal vessels caused by the vascular obstructions induced by the PRP^24^. However, in contrast to the increase of the SFCT, the choroidal MBR was reduced at this time. The difference between the increased SFCT and the decreased choroidal flow would suggest the presence of hemostasis in the subfoveal choroid.

Some studies have reported that the choroid was significantly thinner following PRP in eyes with S-NPDR relative to untreated eyes with S-NPDR in the later times after PRP^8,14,15^. Our results showed that the SFCT was decreased after 8 weeks post-PRP. These results indicate that the SFCT increased transiently in the very early period after PRP but decreased at a later time.

It has been reported that photocoagulation essentially destroyed the choriocapillaris^14,25,26^, and the heat dissipates from the RPE to the adjacent outer retina^24^. Thus, a large number of these cells are destroyed after the many photocoagulation burns by PRP. Taken together, it is most likely that the choroidal blood flow is decreased after PRP treatment at a later time because those choroidal damage would result in a failure of the choroidal vasculature to re-perfuse or reorganize.

The percentage reduction of the choroidal MBR and the SFCT at Week 12 relative to that at the baseline was approximately 86 and 94% in the conventional laser group. Similarly, the percentage reduction was reportedly to be 70 and 86% of the baseline in non-diabetic subjects at approximately 9 years after the PRP^8^. These findings suggest that the reduction of chodroidal blood flow and the SFCT continued to decrease for a long time after the PRP by the conventional laser.

The effects of the photocoagulation was not limited to the lesioned area but extended outside the area by at least 1 to 2 mm after the conventional laser treatment^14^. The PASCAL treatment has several advantages over conventional laser therapy including the short-pulse duration which decreases the width and the axial extent of the retinal burns of the RPE and outer retinal layer^19^. The efficacy of PASCAL treatment, however appears to be less than conventional laser therapy when the same number of laser spots are made^27^. Muqit *et al*. have reported that PDR requires 3998 PRP burns and an area of 456 mm^2^ and severe PDR requires 6924 PRP laser burns in an area of 836 mm^2^ to achieve a complete regression of the disorder^28^. Therefore, we performed a much larger number of burns with a mean of 4959 burns in the PASCAL treatment group than in the conventional group with a mean of 1524 burns.

Our results showed that the choroidal MBR and SFCT were significantly reduced during the 12 weeks after PRP by PASCAL. In addition, there were no significant differences in the percentage reduction of the choroidal MBR and the SFCT between the conventional laser and PASCAL treatment groups throughout the post-PRP period. We are not certain how deeply and accurately the choroidal blood flow are measured in each individual. This could influence the results and the non-significant correlations with all parameters in this study. However, it has been reported that the choroidal blood flow determined by LSFG was significantly positively correlated with SFCT and the luminal area in a larger number of normal eyes^29^. These results imply that LSFG with the 830-nm wavelength can measure whole choroidal vascular layers including Sattler’s layer and Haller’s layer. No significant differences between the conventional laser and PASCAL treatment groups indicate that the PASCAL therapy with sufficient number of photocoagulation burns can lead to a similar level of reduction of the choroidal blood flow and morphological changes as the conventional laser for at least 12 weeks after the PRP. One advantage of the PASCAL treatment is that the photocoagulation spot will not expand because of the short-pulse durations, this might then cause some differences in the choroidal blood flow and the morphological changes in the later period after PRP.

There are several limitations in this study. First, we used a small area centered on the fovea to measure the choroidal MBR, and the width of the binarized region was 1500 μm surrounding the fovea. This indicates that the choroidal measurements were performed only in the center and not in the lesioned area or in the entire choroid. Second, the measured morphological area and the area that was used to assess MBR by LSFG were not the same. It would be better to measure and compare these values using 3D OCT scans for the same region that was used to assess MBR by LSFG. Third, we evaluated the chodoidal blood flow and morphological changes until 12 weeks after PRP. Thus, it is not known whether the choroidal blood flow and SFCT gradually decreased after 12 weeks. Fourth, the number of the patients was not large. Fifth, the SFCT before treatment in the patients was different, and it would be better to enroll patients with similar SFCT to evaluate the choroidal blood flow and SFCT. However, it is not easy to enroll S-NPDR patients with similar choroidal thickness. Sixth, one eye had a temporal macular edema in each of the conventional laser group and the PASCAL group. The MBR in the macular region originates mainly from the choroid with approximately 92% of the choroidal circulation. Accordingly, the effect of ME development after PRP on MBR value is probably limited. Further longitudinal studies using a larger number of subjects with similar SFCT and 3D scans will be necessary for clarification.

In conclusion, the choroidal MBR and the SFCT are significantly reduced during the 12 weeks after completion of PRP after both conventional laser and PASCAL therapy. Our results suggest that sufficient PRP treatment performed by the PASCAL method reduced the choroidal blood flow and the choroidal components. The change in choroidal MBR is not reflected in the choroidal morphology in the early treatment period.

## Methods

### Ethics statement

This was a prospective, cross-sectional, single-center study, and the procedures used were approved by the Ethics Committee of the Nagoya University Hospital (Nagoya, Japan) and registered with the University Hospital Medical Network (UMIN)-clinical trials registry (UMIN UMIN000028453)(31/Jul/2017). The protocol of this study conformed to the tenets of the Declaration of Helsinki. The nature of the study was explained to all patients, and a signed informed consent was obtained.

### Subjects

This study was conducted at the Nagoya University Hospital from July 2016 through December 2017. Patients who met the inclusion and exclusion criteria were randomly assigned to conventional laser treatment or PASCAL treatment by an independent investigator (Y.U.) with an allocation ratio of 1:1. In addition, patients who did not have any treatments for 12 weeks were enrolled in this study as the control group.

All patients had a comprehensive ophthalmic examination including measurements of the intraocular pressure (IOP) and axial length, slit-lamp examinations, fundus examinations, and OCT before and 1, 4, 8, and 12 week(s) after the PRP treatment. We asked all patients to abstain from alcoholic and caffeinated beverages on the morning of the day of the examination, and dilated the pupil 30 min before the examinations with tropicamide phenylephrine eye drops (Mydrin-P, Santen Pharmaceutical, Osaka, Japan). Before the examination, the subjects rested for 10 to 15 min in a quiet dark room, and all examinations were performed in the sitting position at approximately noon to avoid diurnal variations^30–32^.

The IOP was measured with a handheld tonometer (Icare; TiolatOy, Helsinki, Finland), and the axial lengths were measured by partial optical coherence interferometry (IOLMaster; Carl Zeiss Meditec, La Jolla, CA). The diastolic blood pressure (DBP) and the systolic blood pressure (SBP) were measured with an automatic sphygmomanometer (CH-483C; Citizen, Tokyo, Japan). The mean arterial blood pressure (MAP) and mean ocular perfusion pressure (MOPP) were calculated as follows: MAP = DBP + 1/3(SBP-DBP), and MOPP = 2/3MAP − IOP.

### Exclusion Criteria

The exclusion criteria included the presence of vitreous haemorrhage, severe cataract, history of intraocular surgery or photocoagulation, retinal or choroidal pathology, e.g., age-related macular degeneration, choroidal neovascularization, vitreomacular traction, epiretinal membrane, macular hole, and medical conditions that could influence the haemodynamics of the eye other than diabetes, such as hypertension, arrhythmia and vascular diseases.

### Panretinal Photocoagulation Parameters

PRP was performed in the eyes with S-NPDR according to the recommendations of the Early Treatment Diabetic Retinopathy Study group^33^. Fluorescein fundus angiography (FA) showed that all patients had non-perfused retinal areas in three or more quadrants. The PRP was delivered through a widefield contact lens (Ocular Mainster PRP 165; Ocular Instruments, Bellevue, WA) using a slit-lamp-adapted photocoagulator. Topical anesthesia was used on all eyes.

All PRPs were performed by the same surgeon (YM). A spot size of 200 µm was used for both types of PRPs. A pulse duration of 200 ms was used with the conventional laser and 20 ms with the PASCAL protocol. In the conventional laser group, PRP was performed on the inferior, nasal, superior, and temporal quadrants in that order at an interval of 1 week between treatments.

The laser treatments were performed with a 532-nm wavelength argon laser device (NOVUS Varia; Lumenis, San Jose, CA, USA). To create an effective retinal burn, the power was set at 170 to 230 mW, and the exposure was maintained until a grayish-white lesion was seen. The spots were placed at 1 spot distance apart in the conventional group. The number of burns applied during each session ranged from 300 to 400 spots and a total of 1200 to 1600 burns were applied to each eye.

With the PASCAL protocol (PASCAL SlimLine, Topcon, Tokyo, Japan), a 5 × 5 square grid was used to treat 25 spots simultaneously. PRP was performed in two sessions at precisely 1 week between the treatments, and the power was set to 300 to 650 mW.

### Laser speckle flowgraphy (LSFG)

The LSFG-NAVI device was used to determine the relative choroidal blood flow. The principles of LSFG have been described in detail^34^. To evaluate the choroidal blood flow in the macular region, the center of a square (250 × 250 pixels, 6.31° × 6.31°) was centered on at the fovea (Fig. 1A). The MBR determined by LSFG in the macular region originates mainly from the choroid with approximately 92% of the sum of the choroidal and retinal circulation^35^. The measurements have excellent reproducibility with a coefficient of variation (COV) of 4.7 for the choroidal blood flow^36^. The LSFG was performed three times at each time-point in all eyes, and the average of the values was used for the statistical analyses.

### Measurements of choroidal thickness and differentiation of luminal and stromal areas

SD-OCT (Spectralis OCT, Heidelberg Engineering, Heidelberg, Germany) was used to obtain the choroidal images. The choroidal thickness was measured with the SD-OCT using the enhanced depth-imaging (EDI) technique. The SFCT was measured manually using the vertical and horizontal scanned SD-OCT images as the distance from the hyperreflective RPE line to the choroid-sclera border with the calliper tool on the SD-OCT device (Fig. 2). The choriocapillary layer thickness was measured perpendicularly as the distance from the outer edge of the hyperreflective RPE to the innermost point of the large choroidal vessel^37^. The mean of the SFCT in the vertical and horizontal SD-OCT images was calculated. Two clinicians who were masked to the other findings measured the SFCT and the mean of values was used for the statistical analyses.

The EDI-OCT images were analysed using the ImageJ software (ImageJ version 1.47, NIH, Bethesda, MD). The examined region was 1500 μm wide in the subfoveal choroid, and it extended vertically from the RPE to the chorioscleral border using horizontal SD-OCT image (Fig. 3). The binarization of the choroidal area in the EDI-OCT image was done with a modified Niblack’s method^38^. The light areas were defined as the stromal areas and the dark areas the luminal areas. After adding the data of the distance between the pixels, the luminal and interstitial areas were automatically calculated. Two clinicians who were masked to the other findings measured the areas and the mean was calculated.

### Statistical analyses

The sample size was calculated according to the choroiidal MBR determined by LSFG in previous studies that described an approximately 20% reduction of the choroidal MBR after traditional PRP^8^. At 90% power and an α level of 0.05, a supposition that the mean reduction of the choroidal MBR after PRP is 20% would require 17 participants in each group, based on the reported standard deviation. Assuming a dropout rate of approximately 20%, 21 patients in each group needed to be enrolled.

The choroidal MBR, SFCT, choroidal area, luminal area, stromal area and IOP, and MOPP were determined before and 1, 4, 8, and 12 week(s) after the PRP treatment. The correlations of the SFCT with the other factors were determined by a mixed model method. The mixed model was used for analysing the dynamic longitudinal data because it can describe each individual’s pattern of change even in the presence of missing data points^39^. Therefore, we used a mixed model to incorporate the appropriate covariates between repeated measured values over time.

We used the following equation:

$${{\rm{y}}}_{{\rm{ij}}}={a}_{i}+f({t}_{j},\,{G}_{i}:b)+{\varepsilon }_{ij}$$

i(subject) = 1, …, 17 in the conventional laser group and 23 in the PASCAL group, j(time) = 0, 1, 4, 8, 12 where yij is the variable at time j on subject i: *α*_*I*_ is a subject-specific random effect; *Gi* = 1 represents the affected eye and 0 the unaffected eye. The function f(*tj*: *b*) represents a fixed effect of time on the variables. For the residual term e_ij_ of the values, we assumed a heterogeneous compound symmetry structure within patients.

The agreements between the observers for quantitative analysis of the SFCT and choroidal area was analyzed using intraclass correlation coefficient (ICC) statistics. All statistical analyses were performed with SAS9.4 (SAS Inc., Cary). A *P* value < 0.05 was considered statistically significant.
